# A Case Report of Femoral Hematoma following Obturator Nerve Block

**DOI:** 10.1155/2021/2556645

**Published:** 2021-11-13

**Authors:** Kiyoshi Moriyama, Kumi Moriyama, Tomoki Kohyama, Kunitaro Watanabe, Mieko Chinzei, Tomoko Yorozu

**Affiliations:** ^1^Department of Anesthesiology, Kyorin University School of Medicine, Mitaka, Japan; ^2^Sakuramachi Hospital, Koganei, Japan; ^3^Hino Municipal Hospital, Hino, Japan

## Abstract

**Background:**

When an obturator nerve block (ONB) is performed, the conventional landmark method or ultrasound-guided method is used. The major complications of this block are hematoma, but there are very few reports of its complications. We encountered massive bleeding and a huge hematoma after ONB. *Case Presentation*. A 95-year-old female underwent transurethral resection of the bladder tumor. Induction of anesthesia was accomplished via spinal anesthesia and right ONB using the landmark method. Postoperatively, subcutaneous bleeding was detected in the lower right interior thigh. Concentrated red cell transfusion was conducted to address the anemia. There was no subsequent expansion of the hematoma. It resolved on postoperative day (POD) 53. The hematoma was deemed to be inadvertently introduced due to an obturator artery puncture during the obturator nerve block.

**Conclusions:**

Close attention is necessary to avoid advancing the needle too deep into the obturator during obturator nerve block.

## 1. Introduction

Obturator nerve block has been widely performed to reduce the risk of bladder wall perforation during transurethral resections of the bladder and currently for major knee surgery in addition to femoral and sciatic nerve blocks [1]. When an obturator nerve block (ONB) is performed, the conventional landmark method or ultrasound-guided method is used. The major complications of this block are hematoma, but serious bleeding following peripheral nerve blocks is rare [2]. We encountered massive bleeding and a huge hematoma after ONB.

## 2. Case presentation

This is a case of a 95-year-old female, 140 cm tall, weighing 42 kg. She underwent artificial head replacement for a fracture of the right femoral neck when she was 92 years old. Other pertinent history included traumatic subdural hemorrhage and hypertension. Her current medical history included asymptomatic gross hematuria, and transurethral resection of bladder tumor (TUR-BT) was scheduled to closely examine a papillary bladder tumor. Preoperative examination showed anemia with a hemoglobin of 8.2; hence, 2 units of red blood cells were transfused. Coagulation function was normal, but the D-dimer level was elevated at 2.29. Ultrasound of the lower leg was performed, and DVT was noted in the lateral branch of the left soleus vein. No anticoagulant therapy was administered due to the anemia as a sequela of the bladder tumor.

Spinal anesthesia and right obturator nerve block were planned to resect the tumor close to the right ureteral ostium. Spinal anesthesia was performed using a 25-gauge Quinke needle from L3/4, and 2.4 mL of 0.5% high specific gravity bupivacaine was injected. Right obturator nerve block was performed using a Sonokolect Needle™ (manufactured by Hakko Co., Ltd., Medical Device Division) using a nerve stimulator. Puncture was performed 2 cm to the right and 2 cm caudal to the pubic symphysis, and twitch of adductor muscles at 0.5 mA was observed at the 3^rd^ attempt. 20 mL of 1.5% mepivacaine was injected after negative aspiration. Surgery time was 50 minutes, and the anesthesia time was 81 minutes.

**Figure 1:**
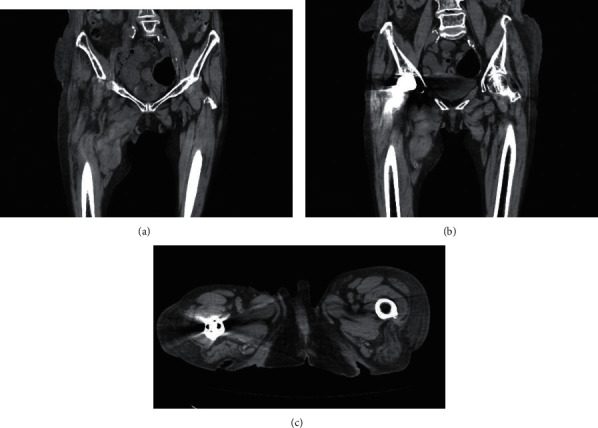
(a) CT on POD 4 showing hematoma spreading from the front of the right pubis to the inner aspect of the thigh. (b) CT on POD 9. Hematoma progression was observed without expansion. (c) CT on POD 53. The hematoma and subcutaneous bleeding disappeared.

On postoperative day (POD) 1, subcutaneous hemorrhage was observed on the inner aspect of the lower right thigh. Hemoglobin decreased from 8.2 to 6.7; therefore, 2 units of RBC were transfused. Postoperative urine color was pale yellow, and no bladder bleeding was demonstrated. On POD 2, swelling was observed from the anterior aspect of the right inguinal region to the inner aspect of the thigh and the popliteal fossa. Contrast-enhanced CT performed on POD 4 revealed a hematoma spreading anteriorly from the right pubis to the inner aspect of the thigh (Figure 1(a)). Hemoglobin was still low, and another RBC unit was transfused. Thereafter, the hematoma and subcutaneous hemorrhage advanced to the medial thigh to the popliteal fossa extending to the back of the lower leg, and hematoma did not expand on POD 9 (Figure 1(b)). To reassess the hematoma, a follow-up blood test and CT were conducted, and there was no exacerbation. She was discharged on POD 17. The hematoma and subcutaneous bleeding disappeared on POD 53 (Figure 1(c)).

## 3. Discussion

It was suggested that the hematoma spread from the obturator artery puncture site during obturator nerve block. In this case, bleeding from the bladder area after surgery was considered as the hemoglobin level decreased from 8.2 to 6.7. However, the urine color was clear, and bladder bleeding was ruled out. Hematoma and swelling of the right femoral area developed, suggesting the cause of anemia. Bleeding from the site of the femoral neck fracture surgery was proposed. We needed MRI to obtain information about the femoral bone and the instrument; however, due to the nature of the imaging study, MRI was deferred due to the instrument and an enhanced CT was performed instead. Her kidney function was not good, so we reduced the dose of medicine and performed CT. Bleeding was estimated to be approximately 600 mL. The causes of massive bleeding and hematoma are discussed in three ways: method, anatomy, and patient background.

First, there are two classic pudendal approaches: the landmark method and the ultrasound-guided block method [3, 4]. The advantages of the landmark method include lower costs, shorter procedure time, and higher success rates. Disadvantages include the following: it requires skill, and if the needle is swung too much on the head side, there is a risk of intraperitoneal puncture, and attention must be paid to avoid puncture of the closed artery and vein accompanying the obturator foramen. On the other hand, the ultrasound-guided method has several advantages. It can be performed relatively easy and successfully even by a novice. Moreover, it has a good safety profile because it does not enter the obturator hole. Because vessels are easily identified with ultrasound, the incidence of vascular puncture may be reduced, but complete prevention cannot be assumed [5]. Disadvantages include an additional cost for ultrasonic device. As the peripheral obturator nerve anatomy varies among individuals and the success rate is not always high, using dual guidance is a good and safer option. Recently, the ultrasound-guided block was performed more often, but some anesthesiologists prefer to do the block using the landmark methods. We need to realize that a blinded technique may cause accidental puncture of the artery.

Second, in addition to the method, this case includes age-related problems. The elderly have less muscle than the young, and their arterial walls are vulnerable. If arterial puncture occurs accidentally, arteries are prone to tear. Muscle around the artery provides protection to the artery and prevents bleeding expansion. However, due to the decreased muscle mass of the elderly population, the bleeding progresses rapidly. Bleeding and hematoma are the major complications of ONB, but there have been no case reports of massive bleeding with ONB. Currently, we induct anesthesia (including blocks) in elderly patients, so the risk is likely to increase. Third, due to anatomical problems, the obturator artery is accompanied by the obturator nerve within the obturator foramen and penetrates the external obturator muscles and exits the obturator foramen. However, the obturator artery has many exceptions, and if the needle is advanced deeply into the obturator foramen, it can be said that the area is at high risk of vascular puncture.

## 4. Conclusions

We encountered a case of massive arterial bleeding requiring transfusion therapy due to obturator artery puncture during nerve block. When performing the obturator nerve block using the classic genital approach landmark method, we need to perform the procedure very carefully not to advance the needle too deep into the obturator foramen. If the landmark method cannot stimulate the adductor muscle, it is better to change the method to ultrasound-guided nerve block.

## Data Availability

Data are available on request to the corresponding author, Kiyoshi Moriyama, via e-mail.

## Abbreviations

ONB: Obturator nerve block
VTE: Venous thromboembolism
TUR-BT: Transurethral resection of bladder tumor
DVT: Deep-vein thrombosis
POD: Postoperative day.
